# Cross-Cultural Validation of the Renzi Score for Obstructed Defecation Syndrome

**DOI:** 10.7759/cureus.79058

**Published:** 2025-02-15

**Authors:** Yara Nakhleh Francis, Wasef Na'amnih, Amir Farah, Jawad Karram, Ala Aiob, Lior Lowenstein, Aied Abu Zhaya, Amir Mari, Susana Mustafa Mikhail

**Affiliations:** 1 Obstetrics and Gynecology, Galilee Medical Center, Nahariya, ISR; 2 Azrieli Faculty of Medicine, Bar-Ilan University, Safed, ISR; 3 Epidemiology and Preventive Medicine, School of Public Health, Faculty of Medicine, Tel Aviv University, Tel Aviv, ISR; 4 Surgery, Medical College of Wisconsin, Milwaukee, USA; 5 Obstetrics and Gynecology, EMMS Nazareth Hospital, Nazareth, ISR; 6 Gastroenterology and Hepatology, EMMS Nazareth Hospital, Nazareth, ISR

**Keywords:** obstructed defecation syndrome (ods), renzi score, sacrocolpopexy, surgery, urogynecology

## Abstract

Background

The Renzi obstructed defecation syndrome (ODS) score is a validated and published method used for the diagnosis and staging of patients with ODS. It consists of a five-item score with a total score ranging from 0 to 20. This study aimed to cross-culturally validate the Renzi ODS score by translating it into Arabic.

Methods

This prospective study included 30 women who had undergone sacrocolpopexy for pelvic organ prolapse repair during the years 2021-2023. The Renzi ODS score was professionally translated from English to Arabic by native speakers, constructed, and validated by five professional and experienced individuals in the study domain for adequate content validity. Data were obtained through a face-to-face interview at baseline and by telephone four weeks later. Cronbach’s alpha coefficient was calculated to determine the homogeneity of the items, item-total, and item-item correlations, and to assess the impact of each item on internal consistency separately. A simple kappa coefficient for each item was assessed to evaluate test-retest reliability.

Results

A total of 30 women aged 42-74 years who had undergone sacrocolpopexy were included in this study. Overall Cronbach’s alpha coefficient for all items indicated that the five items have enough homogeneity in the Renzi ODS score. Inter-item correlations showed a high degree of correlation across all items, which indicates a strong association between each item and the total score of the Renzi ODS. For test-retest reliability, results indicated good to excellent reliability of the Renzi ODS score.

Conclusions

This study validated the Arabic Renzi ODS score, confirming its role as a reliable and consistent tool for evaluating ODS in patients after sacrocolpopexy.

## Introduction

Sacrocolpopexy has long been considered the gold-standard procedure for women with apical prolapse [1]. In recent decades, the procedure has predominantly been performed using laparoscopic or robotic-assisted techniques. Evidence suggests that these minimally invasive approaches offer success rates comparable to traditional open abdominal sacrocolpopexy while providing additional advantages such as reduced blood loss, shorter hospital stays, and faster recovery times [2,3].

Despite its high success rate, sacrocolpopexy is associated with various complications. These include functional impairments such as voiding dysfunction (18%), dyspareunia (8%), and bowel dysfunction, which has been reported in 10% to 50% of cases [4].

Bowel dysfunction following laparoscopic sacrocolpopexy remains an underexplored area, despite recognized symptoms such as constipation, obstructed defecation syndrome (ODS), and dyschezia. Given that studies have reported an increase in ODS following abdominal sacrocolpopexy, it is plausible that similar issues may arise after laparoscopic sacrocolpopexy. While extensive literature exists on urinary dysfunctions and strategies for their prevention, the prevalence and underlying mechanisms of bowel dysfunctions following sacrocolpopexy remain poorly understood [5,6].

The Renzi ODS score is a simple, validated tool used for diagnosing and staging ODS. This five-item scale assigns total scores ranging from 0 (no symptoms) to 20 (severe ODS), with a cutoff of ≥9 demonstrating high sensitivity (92%) and specificity (96%) in distinguishing ODS patients from healthy individuals [7]. A study on postpartum constipation using the Renzi ODS score found significantly higher scores among women who had undergone vaginal delivery compared to those who had cesarean sections [8]. Additionally, studies in urogynecology have demonstrated reduced postoperative constipation using the Renzi ODS score in patients undergoing natural orifice transanal endoscopic rectopexy for complete rectal prolapse and natural orifice endosonographic colposuspension with rectopexy [9].

This study aims to validate the application of the Renzi ODS score by translating it into Arabic to assess the prevalence of ODS following sacrocolpopexy. This adaptation will facilitate its broader application in Arabic-speaking countries, improving the evaluation and management of ODS in women undergoing sacrocolpopexy.

## Materials and methods

Study design and population

A prospective study was conducted on 30 women at the Urogynecology and Pelvic Floor Unit of the EMMS Nazareth Hospital, in Nazareth, Israel, which is home to 46% of Israel’s Arab population. The EMMS Nazareth Hospital is a regional teaching hospital and is the district’s main trauma center that serves a population of over 250,000 people.

Inclusion criteria were women aged 18 years or older who had undergone sacrocolpopexy surgery between September 2021 and November 2023.

Study tools and data collection

After obtaining approval from the original developers of the ODS score, the Renzi ODS score was professionally translated from English to Arabic by the study authors who speak both languages natively, constructed, and validated by five professional and experienced individuals in the study domain for adequate content validity. The study tool is an internationally validated questionnaire. The validation was conducted for each of the five items in the questionnaire: excessive straining; incomplete rectal evacuation; use of enemas and laxatives; vaginal-anal-perineal maneuvers to attempt defecation; and abdominal discomfort/pain. Data for all co-variates were obtained through a face-to-face interview at baseline; however, in the second phase, women were contacted by telephone for a second Renzi score application after four weeks. The feasibility of the questionnaire was tested among a small sample (five interviews) and the quality of the translated questions was examined and discussed by experts in the field, including an assessment of face validity.

Cronbach’s alpha coefficient was calculated to determine the homogeneity of the items, item-total, and item-item correlations, and to assess the impact of each item on internal consistency separately.

A second interview was conducted after four weeks to evaluate the Renzi ODS score's reliability. It was independent from the first, as neither the patients nor the researchers were aware of the scores achieved in the first evaluation. This time interval was considered appropriate since it is long enough to prevent recall bias and short enough to ensure that there were no changes in the construct to be measured [10]. Since it is an ordinal score, the simple kappa coefficient for each item was assessed to evaluate test-retest reliability [11]. Kappa is “acceptable” when it is >0.40, “fair to good” if it is between 0.40 and 0.75, or “excellent” if it is >0.75 [12].

Physical examination to assess pelvic organ prolapse (POP) using the Pelvic Organ Prolapse Quantification (POP-Q) system was performed by a urogynecologist at the first visit. The POP-Q system is a standardized method used to assess and quantify the degree of POP. The POP-Q system involves a physical examination where specific points in the vagina are measured relative to the hymen. These measurements provide an objective way to describe the extent of prolapse. Point Bp is located on the posterior vaginal wall, midway between the hymen and the vaginal cuff or apex. The position of the point Bp is measured in centimeters relative to the hymen. A negative value (e.g., -3 cm) indicates that the point is positioned above the hymen and within the vaginal canal, reflecting no or minimal prolapse. A positive value, however, indicates that the point has descended past the hymen, with the number representing the distance below the hymenal plane.

The measurement of the point Bp is crucial for diagnosing and staging posterior compartment prolapse, often referred to as rectocele [13], which can be associated with ODS. These pelvic organ abnormalities can affect the coordination and function required for normal defecation, leading to symptoms such as a feeling of incomplete evacuation, excessive straining, and the need for manual assistance during bowel movements [14,15].

Statistical analysis

A factor analysis was performed to identify underlying constructs in the survey items and to build scores. The scree plot of eigenvalues was used to determine the number of components. Cronbach's alpha was used to assess internal consistency across the items that were included in these scales. The associations between Bp physical examination and Renzi ODS score were examined using Spearman’s correlation coefficients. The test-retest reliability was determined by the intraclass correlation coefficient (ICC) and simple Kappa coefficient. All statistical tests were two-sided, and p < 0.05 was considered statistically significant. Data analysis was performed using SPSS version 28 (IBM Corp., Armonk, NY).

Ethical approval

All procedures were performed according to local guidelines and regulations. The questionnaire was filled out anonymously. The participants were given a detailed explanation of the study in their native language and were asked to provide informed consent before participation in the study.

## Results

A total of 30 women aged 42-74 years (mean = 59.9 (SD = 9.2); median = 63 (IQR = 14.0)) who had undergone sacrocolpopexy surgery were referred to the Urogynecology and Pelvic Floor Unit at the EMMS Nazareth Hospital and were included in this study. The median levels of Bp and Renzi ODS scores in the first and second meetings were -2.0 (IQR = 1.0), 5.0 (IQR = 9.2), and 4.5 (IQR = 8.0), respectively (Table 1). Table 2 displays the Cronbach’s α coefficient calculated for the five items in the first and second meetings and when deleting each item separately to assess internal consistency. The overall Cronbach’s α coefficient for all items in the first and second meetings were 0.704 and 0.677, respectively, which indicated that the five items have enough homogeneity in the Renzi ODS score (Table 2). A range of 0.52 to 0.73 that was calculated considering deleting each item separately indicated that excluding any item would not significantly influence reliability (Table 2). Inter-item correlations were <0.75 in the first meeting and <0.70 in the second meeting, thus there is no redundancy between the items (Table 2).

**Table 1:** Test-retest reliability of the ODS first-second meeting (n = 30). ODS: obstructed defecation syndrome; ICC: intraclass correlation coefficient.

Item	Kappa coefficient	Mean (SD)	Item-total correlation	ICC (95% CI)
1. Excessive straining	0.68	3.43 (2.92)	0.91	0.95 (0.89-0.98)
2. Incomplete rectal evacuation	0.60	2.83 (2.68)	0.76	0.87 (0.72-0.94)
3. Use of enemas and laxative	0.59	0.40 (1.00)	0.70	0.80 (0.58-0.91)
4. Vaginal-anal-perineal maneuvers to attempt defecation	0.70	1.57 (2.75)	0.89	0.94 (0.88-0.97)
5. Abdominal discomfort/pain	0.71	2.80 (2.72)	0.86	0.92(0.83-0.96)

**Table 2:** Frequencies for age, point Bp physical examination, and Renzi ODS score in the first and second meetings. ODS: obstructed defecation syndrome; SD: standard deviation; IQR: interquartile range; min: minimum; max: maximum.

	Mean (SD)	Median (IQR)	Range (min-max)
Age (years)	59.9 (9.2)	63.0 (14.0)	32.0 (42.0-74.0)
Point Bp value	-2.23 (1.0)	-2.0 (1.0)	5.0 (-3.0-2.0)
Renzi ODS score - first meeting	5.7 (4.5)	5.0 (9.2)	13.0 (0.0-13.0)
Renzi ODS score - second meeting	5.3 (4.3)	4.5 (8.0)	13.0 (0.0-13.0)

**Figure 1:**
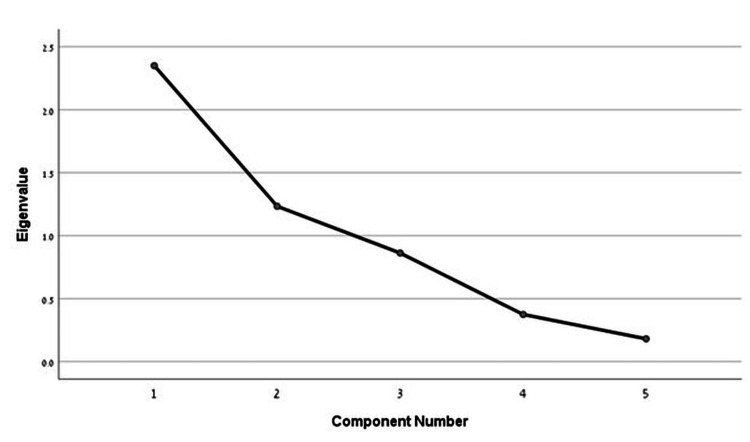
Scree plot of eigenvalues to the scale in Bartlett's test of sphericity - first meeting.

Factor analysis for the Renzi ODS score in the first meeting yielded two factors with an eigenvalue > 1, which explained 71.6% of the variance with a Kaiser-Meyer-Olkin index of 0.487, and p < 0.001 in Bartlett's test of sphericity showed that all items are associated to a single construct. However, the three items analyzed have factor loadings >0.5, indicating a high correlation with the principal component (Figure 1).

**Figure 2:**
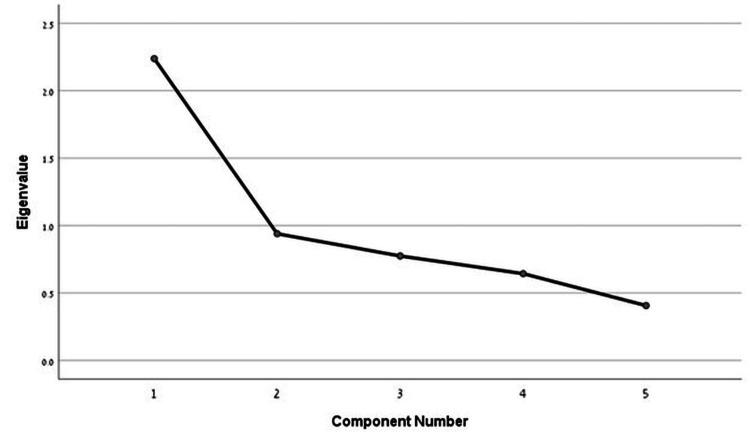
Scree plot of eigenvalues to the scale in Bartlett's test of sphericity - second meeting.

Factor analysis for the Renzi ODS score in the second meeting yielded one factor with an eigenvalue > 1, which explained 44.8% of the variance with a Kaiser-Meyer-Olkin index of 0.715, and p = 0.01 in Bartlett's test of sphericity showed that all items are associated to a single construct. However, four items analyzed have factor loadings >0.5, indicating a high correlation with the principal component (Figure 2).

A high degree of correlation was demonstrated across all items, ranging from 0.70 to 0.91, which indicates a strong association between each item and the total score of the Renzi ODS. Among the individual items, items 1 and 3 displayed the highest (0.91) and lowest (0.70) item-total correlation, respectively (Table 3). For test-retest reliability, the ICC ranged from 0.80 to 0.95, indicating good to excellent reliability between the first and second assessments of the Renzi ODS. However, quadratic kappa coefficient values for each item ranged from 0.59 to 0.71, which is considered fair to good test-retest reliability of the Renzi ODS (Table 4).

**Table 3:** Cronbach’s alpha coefficient for total Renzi ODS score, item-total correlation, and Cronbach’s alpha with item deleted for each item. Cronbach’s α coefficient for total Renzi ODS score - meeting 1 = 0.704. Cronbach’s α coefficient for total Renzi ODS score - meeting 2 = 0.677. ODS: obstructed defecation syndrome.

Cronbach's alpha coefficient - meeting 1 (n = 30)	Item-total correlation	Cronbach's alpha if the item is deleted
Excessive straining	0.74	0.52
Incomplete rectal evacuation	0.33	0.71
Use of enemas and laxative	0.25	0.73
Vaginal-anal-perineal maneuvers to attempt defecation	0.57	0.60
Abdominal discomfort/pain	0.46	0.66
Cronbach's alpha coefficient - meeting 2 (n = 30)		
Excessive straining	0.61	0.53
Incomplete rectal evacuation	0.38	0.65
Use of enemas and laxative	0.28	0.69
Vaginal-anal-perineal maneuvers to attempt defecation	0.57	0.56
Abdominal discomfort/pain	0.39	0.65

**Table 4:** Spearman’s correlation coefficients determined when comparing point Bp physical examination and Renzi ODS score. ODS: obstructed defecation syndrome.

	Bp	P-value
Renzi ODS score - first meeting	-0.26	0.2
Renzi ODS score - second meeting	-0.51	0.004

## Discussion

Our study validated the Arabic version of the Renzi ODS score in women who had undergone sacrocolpopexy for pelvic organ prolapse.

Bowel dysfunction, including ODS, is a recognized complication that can occur following sacrocolpopexy, particularly in the short term. This may be due to neurological or anatomical changes, as the repositioning and support of pelvic organs during surgery can alter pelvic floor dynamics and rectal function, potentially leading to obstructed defecation. Changes in the pelvic floor’s support structures may disrupt the coordinated movement required for bowel evacuation, resulting in symptoms such as difficulty passing stool or excessive straining.

Currently, there is no universally accepted standard for evaluating ODS in these patients. Various questionnaires are available, but many are lengthy and difficult for patients to complete. The Renzi ODS score was chosen for validation in Arabic because it is a simple and practical tool, consisting of only five clearly defined parameters that patients can easily understand and respond to.

The Renzi ODS score has previously been translated and validated in English and Portuguese [7,16]. Our study found that the Arabic version demonstrates strong internal consistency, as indicated by a high Cronbach’s α coefficient, suggesting that its items reliably measure the same underlying construct. Additionally, low inter-item correlations across assessments confirm that the questionnaire items are not redundant.

Factor analysis of the Renzi ODS score revealed variations in factor structure across different assessments. Initially, two factors were identified, accounting for a significant portion of the variance, though with moderate sampling adequacy and some overlap in item loading. However, in the follow-up assessment, a single dominant factor emerged, explaining a larger proportion of the variance and demonstrating improved sampling adequacy. This suggests a more unified construct over time. Furthermore, most items showed high loadings on the principal component, reinforcing the coherence of the questionnaire.

The Renzi ODS score also exhibited strong item-total correlations, indicating that each item contributes meaningfully to the overall score. Its high test-retest reliability supports the questionnaire’s consistency over time, though some variability in responses was observed, as indicated by fair to good agreement between test administrations. While the Arabic version of the Renzi ODS score is a reliable tool, further refinements could enhance its precision and stability.

From a clinical perspective, postoperative ODS scores in our study were below the established cutoff of 9, which differentiates individuals with ODS from those without. This suggests that after sacrocolpopexy, the women in our cohort did not exhibit obstructed defecation symptoms severe enough to qualify as ODS. However, the lack of preoperative ODS assessments limits our ability to determine whether sacrocolpopexy influenced these scores or if asymptomatic patients were included from the outset. Future studies incorporating both preoperative and postoperative assessments would provide a clearer understanding of the surgery’s impact on bowel function.

Although our study confirms the validity of the questionnaire in Arabic, several limitations must be acknowledged. One potential source of bias stems from differences in data collection methods, as baseline assessments were conducted face-to-face, whereas follow-up evaluations were carried out via telephone. This variation may have influenced participants' responses. Additionally, regional differences in Arabic dialects could affect the consistency of terminology, though the questionnaire was designed using simple and widely understood language to maximize accessibility.

Despite these limitations, our findings demonstrate that the Arabic version of the Renzi ODS score is a valid and reliable tool for assessing ODS in Arabic-speaking populations. Its simplicity and clarity enhance its practical utility, ensuring accessibility across diverse Arabic-speaking communities and improving the accuracy of ODS assessments in clinical and research settings.

## Conclusions

This study successfully validated the Arabic version of the Renzi ODS score, demonstrating its reliability, responsiveness, and interpretability. As a practical and effective tool, it can be widely implemented in clinical practice among Arabic-speaking populations, contributing to improved patient care in the field of urogynecology. Future research should explore its applicability across diverse demographic groups, assess its sensitivity to cultural nuances, and investigate its integration with digital health technologies for broader clinical utility and patient engagement. Additionally, longitudinal studies could further evaluate its predictive value and impact on treatment outcomes over time.
